# Prevalence of mental distress and associated factors among caregivers of patients with severe mental illness in the outpatient unit of Amanuel Hospital, Addis Ababa, Ethiopia, 2013: Cross-sectional study

**DOI:** 10.1186/s40303-015-0014-4

**Published:** 2015-10-08

**Authors:** Mezinew Sintayehu, Haregwoin Mulat, Zegeye Yohannis, Tewodros Adera, Maereg Fekade

**Affiliations:** Department of Nursing, Mekelle University, College of Health Sciences, PO. Box 1871, Mekelle, Tigray region North Ethiopia; Department of psychiatry, College of Health sciences, University of Gondar, Amhara, North Ethiopia; Amanuel mental specialized hospital, Addis Ababa, Ethiopia; Kolfahealth institution, Addis Ababa, Ethiopia

**Keywords:** Caregiver, Mental distress, Associated factors, Severe mental illness, Addis Ababa

## Abstract

**Background:**

Caregivers like family members or other relatives are central and provide not only practical help and personal care but also give emotional support, and they are suffering from plenty of challengeable tasks. These, eventually, cast out family caregivers into multidimensional problems prominently for mental distress like depression, anxiety, sleep problem and somatic disorder which are followed by physiologic changes and impaired health habits that ultimately lead to illness and possibly to death. Numerous studies demonstrate that mental distress of caregivers are two times compared to general populations.

Despite it was not uncommon to observe manifestations of caregivers’ mental distress, yet there was no study on this area. Therefore, this study was intended to assess the prevalence of mental distress and associated factors among the caregivers of persons with severe mental illness in the out patients unit of Amanuel Hospital, Ethiopia.

**Methods:**

Institutional based cross sectional study was conducted from May 1 to 31, 2013 at Amanuel Hospital, Addis Ababa, Ethiopia. Systematic random sampling technique with “k” interval of 13 was employed to withdraw a total of 423 participants from study population. Five psychiatric nurses carried out interview by using standardized and validated Self Reported Questionnaire (SRQ 20). Descriptive statistics, binary and multivariate logistic regression analysis were conducted.

**Results:**

This study revealed that the overall prevalence of mental distress was found to be 221(56.7 %). The factors like missed social support, two or more times admission of patient, care giving for psychotic patient, being farmer and being female were found to be predictors for mental distress of caregivers with this [AOR 95 % CI = 9.523(5.002, 18.132)], 3.293(1.474, 3.3560), 2.007(1.109, 3.634), 2.245(1.129, 4.463) and 3.170(1.843, 5.454)] respectively.

**Conclusions:**

In this respect the study observed that there was a higher level of mental distress experienced by caregivers of patients with severe mental illness in Amanuel Hospital, and social support are strongly associated with mental distress besides to other variables. Effectively planned interventions have to be targeted at alleviating mental distress and actions like on-going psycho-education and mutual support that could expand social support should be implemented in Amanuel hospital health service delivery system.

## Background

### Background and statement of the problems

The burden of mental health problems is increasing globally [1]. It is gradually becoming recognized that mental disorders are a public health problem throughout the world. In 2001, mental disorders accounted for 13 % of the worlds burden of diseases and this figure is projected to increase to 15 % by the year 2020 [2, 3]. Worldwide studies have shown that as many as 450 million people suffer from mental disorder and their disabling effect at individual and national levels to be quite significant. This had led to the recognition, by the member states of WHO in mental health care as one of the priorities and to its inclusion in the program of primary health care [1, 2, 4].

Besides mental disorder, as defined according to diagnostic criteria, the wider concept of mental distress comprises mental disorder as well as other mental problems that may not fall in to standard diagnostic criteria. It refers to a lack of psychological wellbeing affecting a person’s thoughts, feelings, behavior and functioning [5].

Individuals with a severe mental illness have typically been mentally ill for many years and are unable to fulfill daily roles in society normally expected of individuals of their age and intellectual ability; thus, they are most likely receive family care [4].

Available data show that the proportion of persons with severe mental illness living with their relatives ranges between 40 percent in the United States, over 60 % in Africa to more than 90 percent in China [4, 6, 7]. Families not only provide practical help and personal care such as bathing, eating, taking drug but also give emotional support to their relative with a mental disorder in the face of insufficient knowledge, skill to provide care, limited social support and poor mental health facilities [6, 8, 9]. Despite few studies acknowledge positive outcomes for caregivers and makes them feel good and satisfaction about themselves [8, 10, 11], care giving has all the features of a chronic stress experience so well that it is used as a model for studying the health effects of chronic stress [10, 12].

These challengeable tasks, chronic stress, daily hassles and negative caregivers perception bring profound objective and/or subjective burden that involves psychosocial, physical, and financial impact on the caregivers of individuals with severe mental illness which is comparable to that of persons with other illnesses such as Alzheimer’s disease or cancer, especially after deinstitutionalization movement began more than five decades ago because there was transferring of responsibility and day-to-day care to family members [13–15]. As caregivers struggle to balance work, family and care giving, their own physical and emotional health is often ignored. As a result of this and lack of personal, financial, emotional resources and stigma, many caregivers often experience significant physical and mental distress but physical effects of care giving are generally less intensive than the psychological effects [9, 10, 16].

Numerous studies have demonstrated that family caregivers of patients with a severe mental illness suffer from mental distress (especially depression, insomnia, anxiety, somatization, paranoia and obsessive behavior); and often receive inadequate assistance from mental health professionals. Research conducted in British (1992) reported that psychological distress (anxiety, depression, and insomnia) was twice as high as in the general population [17]. Other findings conducted in Latin America and KSA Arab country suggest that 40 % of the caregivers compared to 13 %–18 % of general population and 23.33 % of the caregivers group versus 3.33 % of the control group met the criterion for being at risk of depression for the CES-D 10 scale as they got 10 or greater score respectively [6, 18]. Another study in Nigeria on caregivers of psychiatric out patients reveals almost half of the relatives had psychological distress (43.8 %) [19].

The literature consistently demonstrates that the mental distress of caregivers have been linked to objective burden (like duration and type of care provided, unemployment, duration of illness, behavioral problems, cognitive and functional disabilities of care recipient), subjective burden(such as perceived stigma, negative care giving appraisal, feeling of isolation, anger, sadness, guilty feeling, shame), insufficient social support, age of patient, negative coping mechanism, real stigma, as well as secondary stressors such as finances and family conflict [6, 19, 20].

Generally burden of family caregivers leads to negative consequences not only for themselves but also for patients, other family members, and health care systems [4]. Their negative quality of life has impacted on poor caring, mistreatment or behaving violently to the patients which can cause patients relapse [4, 21]. Burden of family caregivers also causes family conflict and financial problem in individual, family, health care system, distorts the entire family functioning and the families under great stress would give up and reject the mentally ill individuals who would become outcasts socially [9]. Furthermore, as conceptual models of care giving and health suggest that health effects should unfold in a cascading fashion. Caregivers first experience distress and depression, which are followed by physiologic changes and impaired health habits that ultimately lead to illness and possibly to death [22]. However these impacts might be different among caregivers, as the level of burden is related to various factors.

Recently there have been limited studies on caregivers in Africa and most were done in higher income countries. Those researches done in higher income countries regarding the burden, mental and physical effects of caregivers divert the face of the world towards assisting of key caregivers patients with severe mental illness like schizophrenia by developing theoretical model of family intervention like psycho education and social or mutual support and through Early interventions by conducting routine assessments of the depression status of caregivers [6, 9, 22, 23].

In Ethiopia, the practice of psychiatry has evolved over several years spanning from an era of purely traditional practices and beliefs to the modern therapeutic practice of using medications and other therapies. Self-reporting questionnaire (SRQ) is a screening instrument developed by WHO to assess symptoms of mental distress in developing countries. The first study that used SRQ was conducted by kortmann in 1988 who reported a 12 % of mental disorder among adults in Addis Ababa [24]. Later, other investigators assessing community and hospital based samples in Ethiopia reported prevalence estimates from 12 % to 23.9 % [25].

### Significance of the study

Despite all studies done in developed countries, revealed as caregivers of mental ill patients have always been subject to massive input of stresses, burden and mental illness. In Ethiopia, to the best of our knowledge, there was still no published study on this particular issue. Therefore, this study provided the prevalence and factors that associated with mental distress among caregivers of patients with severe mental illness. In addition, this gives important clues for mental health professionals to recognize and offer early interventions by conducting routine assessments of mental distress among caregivers and their available social support, thereby preventing or minimizing mental distress in those caregivers.

The result of this study may help or influence police makers, health managers and planners to formulate national and local mental health policy, and for Amanuel Mental Specialized Hospital to design appropriate service provision by consolidating family therapy which involves ongoing psycho education and mutual support as well. This in turn, might improve the quality of life for patients who are suffering from severe mental illness and further benefit family and the country. In addition to this, it may also serve as base line data for further studies.

### Objectives

#### General objective

To assess the prevalence and associated factors of mental distress among care givers’ of patients with severe mental illness in the outpatient unit of Amanuel Mental Specialized Hospital, Addis Ababa, Ethiopia, May 2013.

### Specific objectives

- To determine the prevalence of mental distress among caregivers’ of persons with severe mental illness
- To identify factors associated with mental distress among caregivers’ of persons with severe mental illness

## Methods

An institution based cross-sectional study was conducted at Amanuel Mental Specialized Hospital, Addis Ababa which serves at inpatient and outpatient bases of people with mental illness and other medical illness. The Hospital has 259 beds including 11 private wing beds and 23 emergency beds and also it has 13 outpatient departments (OPDs). An average of 10,320 patients get service per month and most patients attend the Hospital with family members. The hospital is also playing its pivotal role as a training institute for psychiatric professionals of different levels; so as to expand the service throughout the country by introducing psychiatry service to the primary health care system. Masters of Science degree in integrated clinical and community mental health training program has been one of the programs provided in collaboration with University of Gondar.

An estimation of 4670 people with psychotic disorders which includes schizophrenia and others psychotic disorders; and 880 people with bipolar disorder based on the criteria established in DSM-IV, were thought to be treated per month from previous records in outpatient unit. From these, four hundred twenty three (423) samples were calculated by single population proportion formula by assuming the proportion (p) of mental distress of caregivers to be 50 %, margin of error 5 % with 95 % confidence interval and 10 % of non response rate. Systematic random sampling technique with “k” interval of 13 was employed to withdraw samples from study population. Caregivers were included in the study if their age is greater than 18 years old and gave care for patient more than 6 months. Caregivers were excluded if they have a history of psychiatric disorder before being a caregiver; and professional care giver.

### Data collection technique and tool

Data was collected by using standardized and validated SRQ 20; Oslo-3 (OSS-3); substance use instrument and other socio demographic questions. Five psychiatric nurses were involved to conduct face to face interview of caregivers while they accompanied their care receiver. SRQ 20 instrument was originally developed by the WHO to screen for psychiatric disturbance in primary health care settings in low-income countries. The SRQ is not expected to diagnose mental illness but was designed to indicate mental distress. It is used as a first-stage screening instrument for the second-stage clinical interview. The questions assess about features of common mental disorders, particularly anxiety and depression. SRQ- 20 is now the most widely used version of the instrument. SRQ was supposed to be self-administered, with ‘yes’ or ‘no’ response to each question. However, because of the high illiteracy rate in Ethiopia and other countries of the same status, it has been used in an interview format. This study also was employed interview methods. Respondents were asked about experiencing symptoms of mental distress over the past 1 month. SRQ has been previously translated into Amharic, validated and subsequently used for epidemiological studies in clinical and community settings in Ethiopia. Social support was assessed by using Oslo-3 social support scale (OSS-3). Socio-demographic and other relevant variables about mental distress of caregiver were added to the questionnaire for this study. A structured pretested questionnaire was employed to collect socio-demographic characteristics and other relevant factors of mental distress of caregiver which was adapted from different literature with modification in our cultural context.

### Operational definition

#### Mental distress

SRQ has 20 questions and the score of all questions was combined. The minimum and maximum score are 0 to 20. With cut point 7 and for an individual scored more than 7 was considered as having mental distress.

#### Caregiver

Someone who most giving care and regularly responsible taking care of patients more than other family member or other persons rather than by a professional who is reimbursed for services.

#### Severe mental illness

Comprise both patients with bipolar disorder and psychosis (schizophrenia and other psychotic disorder).

#### Current substance users

When clients used specified substance (for non medical purposes) in the last three months.

#### Ever substance users

When clients used specified substance (for non medical purposes) even once in their lifetime.

#### Missed social support

Scored of 3–8 during Oslo-3 Social Support Scale (OSS-3).

#### Have social support

Scored of 9–14 during Oslo-3 Social Support Scale (OSS-3).

#### Immediate family relative

Son/daughter, and brother or sister.

#### Non immediate family relative

Other relative or non-relative.

### Data management and data quality control issues

Data quality control issues were insured by using validated SRQ 20. Data collectors were trained for three days to use the questionnaire, the ethical principles of confidentiality and data management prior to their involvement with data collection.

Supervisions by supervisors and investigator held regularly during data collection period. The collected data was checked on daily basis for completeness and consistence. Manual and computerized data cleaning was done before data analysis.

### Data processing and analysis

The coded Data was checked, cleaned and entered into epi.INFO 6 version and then exported into Statistical Package for the Social Sciences (SPSS) window version 20 for analysis. Descriptive summary using frequencies, percentage, graphs, median and ranges were used to present study results. Bivariate analysis was done for COR and multivariate analysis was employed to calculate AOR for variables which met *p*-value < 0.2 during bivariate analysis. *P*-value of < 0.05 was considered as statistically significant during multivariate logistic regression.

### Ethical considerations

Ethical clearance was obtained from university of Gondar ethical review board and Amanuel Mental Specialized Hospital. Written Informed consent was obtained from participants and they were informed that participation was on voluntary basis and have full right to withdraw at time of need during the interview process. Moreover, the researchers were striving to protect and to respect the privacy and wellbeing of persons with these conditions. Data that was collected for the purpose of this study was not containing identification information of the participants, thus ensuring the secrecy of the participants.

## Results and discussion

### Description of socio-demographic characteristics

Out of 423 planned respondents, three hundred ninety (390) caregivers of people with severe mental illness were enrolled in this study which gave a response rate of 92.2 %.

### Socio- demographic characteristics and other related factors

From participants, female respondents 214(54.9 %) and married respondents 208(53.3 %) predominates male and never married respondents respectively. The median age of respondents was 42 years old with a range of 65 years old which was highly distributed in the category of > 44 years old 176(45.1 %).

The majority of caregivers have some form of private and governmental job 137(35.1 %) and farmers 83(21.3 %) and half of caregivers earned below 700 birr per month.

Almost three quarters of respondents 291(74.6 %) lived in rural areas, and among the total samples participated 171(43.8 %) had less than or equal to 12 grade while 142(36.4 %) were uneducated and 77(19.7 %) had greater than 12 grade in education profile. Three hundred eleven (79.7 %) were caregivers of patients with psychotic disorders while 79(20.3 %) were caregivers of patients with bipolar disorders. From the total patients almost half of patients 209 (53.6 %) had no history of admission, 95 (24.4 %) had one admission and 86 (21.1 %) had history of two or more admission (Table 1).

**Table 1:** Distribution of socio-demographics characteristics of the respondents among caregivers of people with severe mental illness at Amanuel Mental Specialized Hospital (*n* = 390), Ethiopia, May 2013

Variables	Frequency	Percent (%)
Gender/sex		
Male	176	45.1
Female	214	54.9
Age		
18–24	28	7.2
25–34	82	21
35–44	104	26.7
>44	176	45.1
Marital status		
Single/Never married	73	18.7
Married	208	53.3
Divorce/separated	58	14.9
Widowed	51	13.1
Ethnicity		
Amhara	118	30.3
Tigre	32	8.2
Oromo	123	31.5
Gurage	96	24.6
Others	21	5.4
Religion		
Orthodox Christian	217	55.6
Islam	103	26.4
Protestant	60	15.4
Others	10	2.5
Educational status		
Not educated	142	36.4
≤ Grade 12	171	43.8
> Grade 12	77	19.7
Cont Job (Occupation)		
Employed	137	35.1
Own business	56	14.4
Farmer	83	21.3
Housewives	50	12.8
Jobless	64	16.4
Residence		
Urban	99	25.4
Rural	291	74.6
Average monthly income of the house hold		
≤400 Birr	115	29.5
401–700	114	29.2
701–1200	68	17.4
≥1201	93	23.8
Diagnosis of the patient		
Pychosis	311	79.7
Bipolar disorder	79	20.3
Number of admission		
No admission	209	53.6
One time admission	95	24.4
≥ Two times admission	86	21.1
Duration of care giving in year		
0–5 years	243	62.3
6–10 years	83	21.3
≥11 years	64	16.4

**Fig. 1:**
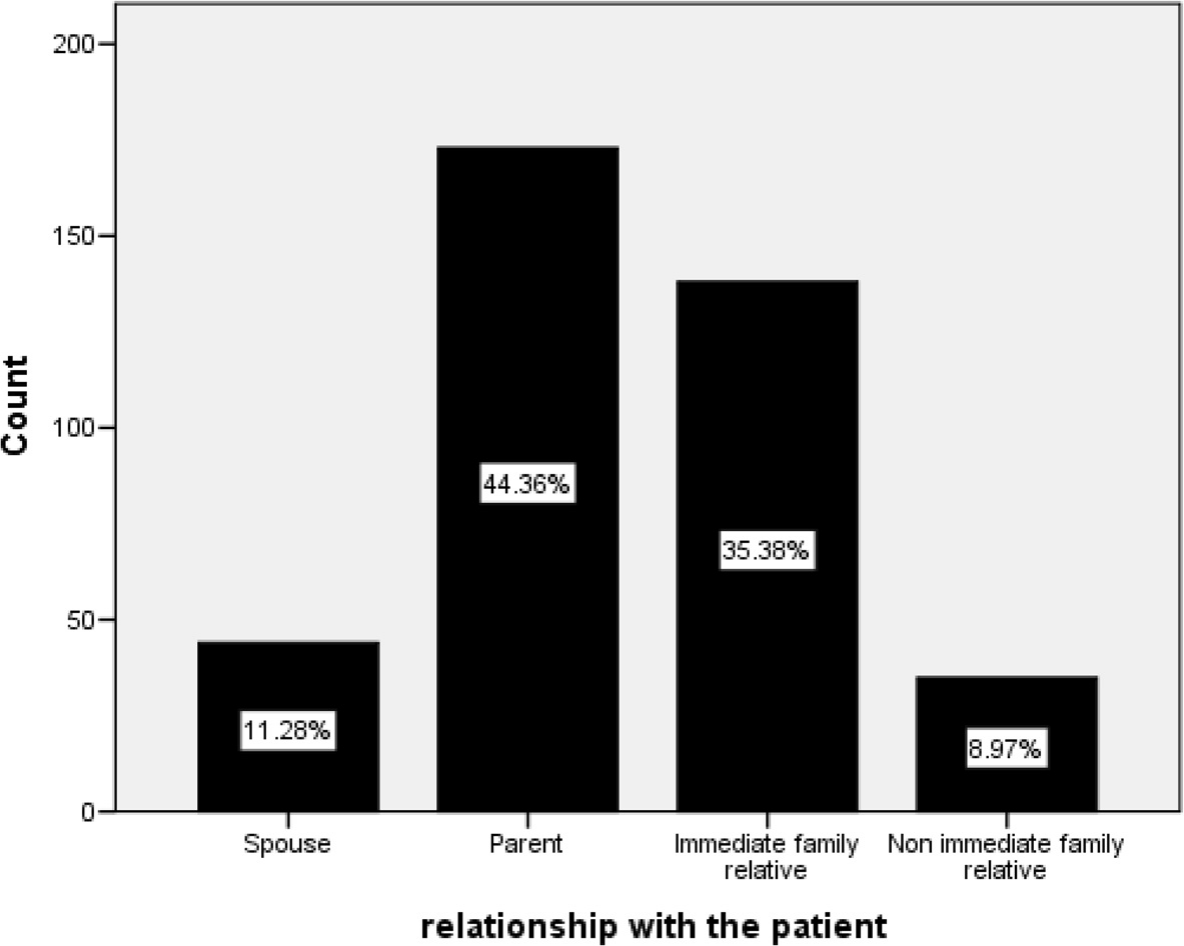
Distribution of caregivers relationship with the care recipient among caregivers of people with severe mental illness at Amanuel Mental Specialized Hospital (*n* = 390), May 2013

Caregivers accompanying patients were mainly parents 173(44.6 %) and immediate relatives 138 (35.4 %) (Fig. 1).

### Substance use history of the respondents

As illustrated in Table 2 out of 390 study subjects 20(5.1 %) were drinking alcohol in the last 3 months, and 194 (49.7 %) had used alcohol once in their life time. Twenty two (5.6 %) of the respondents were using Khat in the last three months, whereas 50 (12.8 %) had practiced Khat chewing at least once in their life time. Six (1.5 %) of the respondents were smoke cigarrate in the last three months while 11(2.8) of respondents had used tobacco products at least once in their life time. Among caregivers in this study, none of them used cannabis in their life time and in the last three months as well. (Refer Table 2. for details).

**Table 2:** Distribution of substance use among caregivers of pople with severe mental distress at Amanuel Mental Specialized Hospital (*n* = 390), Ethiopia, May 2013

Type of substance uses	Ever user history		Within the last three months	
	Freq	Percent (%)	Freq	Percent (%)
Tobaccos				
Yes	11	2.8	6	1.5
No	379	97.2	384	98.5
Alcohol beverages				
Yes	194	49.7	20	5.1
No	196	50.3	370	94.9
Khat				
Yes	50	12.8	22	5.6
No	340	87.2	368	94.4
Cannabis	0	0	0	0
Yes	390	100	390	100
No				

### Assessment of social support of respondents

In the assessment of social support out of 390 subjects using Oslo-3 Social Support Scale (OSS-3), more than three quarter of respondents 297 (76.2 %) scored less than or equal to 8 or who missed social support and the rest 93(23.8 %) had social support that means they were scored 9–14.

### Associated factors of mental distress among caregivers of patients with severe mental illness

In the bivariate analysis, it was shown that marital status, relationship with the patients and educational status were significantly associated with mental distress though they were fail to resist in multivariate analysis with *p*-value < 0.05 and confidence does not include. That was twofold increased in mental distress of divorced/separated and widowed caregivers compared to married persons with odds ratio of [COR 95 % CI = 2.180(1.174, 4.049), 2.146(1.119, 4.114)] respectively. Subjects who were not educated were two fold increased for mental distress compared to those > 12 years education [COR 95 % CI = 2.074(1.176, 3.658)] and parents of caregivers revealed three fold increased to mental distress compared to non-immediate family relative with [COR 95 % CI = 3.270(1.538, 6.952)].

In this study, age > 44 reported higher level of mental distress than in the age interval of 18–24 years old. This difference, however, does not reach statistically significance. In these study subjects other variables like ethnicity, religion, average monthly income, residence and substance use had not relation with mental distress.

Multivariate logistic regression was used to analyze associations between predictors and mental distress which have p value of ≤ 0.2 in bivariate logistic regression, and adjusting for covariates (Table 3). After adjusting for possible covariates, gender, occupation, diagnosis of patients, number of patient admission and social support of caregivers were significant predictors of mental distress among caregivers of patients with severe mental illness with *p*-value < 0.05.

Statistically significant higher rate of mental distress was seen among female caregivers compared to male caregivers (AOR 95%CI = 3.170(1.843, 5.454). Caregivers who were employed appeared to have a decreased risk of mental distress compared to those who were farmers where the risk of mental distress was doubled which is statistical significant [AOR 95 % CI = 2.245(1.129, 4.463). Respondents who took care for psychotic patient had a twofold risk of mental distress than who took care for bipolar disorder patients [AOR 95 % CI = 2.007(1.109, 3.634)]. Those caregivers that cared for patients with one time admission and two or more time admission had experienced two fold and three fold risk for mental distress compared to patients with no admission [AOR 95 % CI = 2.425(1.319,4.459) and 3.293(1.474,3.3560] respectively. Moreover, in this respect the study found a strong relationship between social support and mental distress that caregivers who missed social support were 9 fold risks for mental distress compared to those who had social support. (Refer Table 3 for further information).

**Table 3:** Factors associated with Mental distress of caregiver’s of people with severe mental illness in Amanuel Mental Specialized Hospital, 2013 (*n* = 390)

Socio demographic Variables	Mental distress of care givers			
	yes	no	crude OR (95 % CI)	adjusted OR (95 % CI
Gender/sex				
Male	70	106	1	1
Female	151	63	3.629(2.382,5.531)*	3.170(1.843,5.454)*
Marital status				
Married	105	103	1	1
Single/never married	41	32	1.257(0.735,2.149)	1.768(0.809,3.861)
Divorce/separated	40	18	2.180(1.174,4.049)*	0.992(0.446,2.208)
Widowed	35	16	2.146(1.119,4.114)*	0.662(0.286,1.533)
Educational status				
Not educated	95	47	2.074(1.176,3.658)*	1.083(0.464,2.529)
≤grade 12	88	83	1.088(0.635,1.864)	1.009(0.484,2.103)
>grade 12	38	39	1	1
Cont Occupation				
Employed	73	64	1	1
Own business	23	33	0.611(0.326,1.147)	0.613(0.289,1.300)
Farmer	52	31	1.471(0.842,2.567)	2.245(1.129,4.463)*
House wife	33	17	1.702(0.867,3.341)	1.006(0.436,2.231)
jobless	40	24	1.461(0.796,2.682)	1.695(0.796,3.608)
Relation with the patient	24	20	2.031(0.820,5.029)	1.461(0.476,4.478)
Spouse	114	59	3.270(1.538,6.952)*	1.925(0.762,4.868)
Parent	70	68	1.742(0.813,3.735)	1.515(0.603,3.806)
Immediate family relative	13	22	1	1
Non-immediate family relative				
Diagnosis of the patient	188	123	2.131(1.29,3.518)*	2.007 (1.109, 3.634)*
Psychosis	33	46	1	
Bipolar disorder				1
Number of admission				
No admission	99	110	1	1
One time admission	58	37	1.742(1.063, 2.854)*	2.425(1.319, 4.459)*
≥ Two times admission	64	22	3.248(1.636, 6.448)*	3.293(1.474, 7.356)*
Duration of care giving in year				
0–5 years	129	114	1	1
6–10 years	50	33	1.339(0.807, 2.222)	0.942(0.499, 1.779)
≥11 years	42	22	1.687(0.950, 2.995)	1.271(0.600, 2.693)
Social support of caregiver				
Have no social support	15	78	11.711(6.427,21.560)*	9.523(5.002,18.132)*
Have social support	206	91	1	1
Use of Chat in your life	22	28	0.557(0.306,1.013)	1.109(0.506,2.432)
Yes	199	141	1	1
No				

* refers association *p* value <0.05

### Discussion

In Ethiopia there is no study done on caregivers with severe mental illness. Even researches in middle income countries on mental distress among caregivers of patients with severe mental illness have been minimal and have primarily focused on burden of caregivers. Thus, little is known about mental distress of caregivers of patients with severe mental illness related to their care giving role in developing countries like Ethiopia. This present study was conducted among 390 caregivers of patient with severe mental illness to find out the magnitude of mental distress and associated factors using a cross sectional study design and SRQ 20 instrument. The caregivers were predominantly women (54.9 %), and parents (44.4 %) which were similar to previous studies [6, 9]. The overall prevalence of mental distress for caregivers was found to be 56.67 % that met for being at risk of mental distress (that is score more than 7 on SRQ 20). In Ethiopian community study, the estimated prevalence of general mental distress was between 12 and 23.9 % depending on population [25]. Providing care for patients with severe mental illness places caregivers at higher risk of mental distress compared to estimated community prevalence of mental distress which is more than twice and this is in line with other study reports of developed countries [16].

The prevalence of mental distress among caregivers of patients with severe mental illness according to this study was higher than researches conducted on Nigeria which is almost 50 %, study done on depression in Latino family caregivers and KSA, Arab country have had prevalence rate of 40 % and 23.3 % respectively. The discrepancy observed were because of GHQ tool used in Nigeria, CES-D scale tool among Latino family caregivers, CES-D scale tool and case control study design in KSA Arab country, sample size difference and socio-demographic characteristics of respondents can also be a contributed for the difference.

The demographic characteristic of the caregiver showed that there was a statistically significant association between female gender and risk of mental distress which was in line with finding of many previous studies [6, 11]. However, in some other studies the reverse has been observed [6, 19]. The women are responsible for the emotional care of the children especially in Africa context; it is more acceptable for the woman to take up the role of the caregiver. This may result in low self-esteem and loss of self that may be associated with maternal depression due to subjective care giving burden and other factors like low social status, the affective nature of their responses to stressors and hormonal changes may play their roles [9, 18].

The finding that occupation was significantly associated with risk of mental distress, being employed and worked their own business seems to be protective against mental distress, except for farming. It could partly be explained by; farmers may have been more stigmatized, inaccessible to media/information/lack of knowledge, lower income and exposed to stressful life experiences than those of employed and worked their own business in urban areas. The finding in this study demonstrated that caregiver’s of patients with diagnosis of psychotic disorder experienced higher levels of mental distress than patients with bipolar disorder which was also supported by previous research [9]. This could be explained by; patients with diagnosis of psychotic disorder exhibit more negative or positive symptoms, less functioning and less likely had get improvement than bipolar disorder.

Other factor associated significantly with mental distress among caregivers in this study was number of patient admission which is in agreement with other studies [10]. This indicates frequent relapse and severity of illness which could further exacerbate the caregiver mental distress.

Moreover, this finding provides strong evidence that showed missed social support for caregivers experiencing more mental distress which is keeping with finding of other literatures [9, 11].

In crude analysis, these study also showed that caregivers who had no education, divorced/separated and widowed in marital status and being parent for mentally ill patients experienced higher levels of mental distress than their counter parts with higher level of education, married and non-immediate relatives respectively which were also supported by previous studies despite those variables are failed to maintain their association with mental distress in multivariate analysis [18].

This study found no association between independent variables of caregivers’ age, duration of care giving, ethnicity, religion, resident of caregiver, monthly income and substance use with dependent variable of mental distress, unlike some previous studies where those variable differences were observed [6, 8].

### Limitations of the study

The study design was a cross sectional, hence it provided only a snapshot of the issues faced by the caregivers or it did not permit causal interpretations.

Preexisting mental disorders was not assessed and may have had an influence on the findings.

The variables which were not included were also a factor in the present study for instance the relationship between other stress inducers like positive and negative symptoms of patient, knowledge of caregivers, personal factors, environmental factors and mental distress.

## Conclusions

This finding observed that higher prevalence of mental distress among caregivers’ of people with severe mental illness at Amanuel Mental Specialized Hospital which was 221(56.67 %). poor social support were strongly associated with mental distress which implies caregivers who have not social support have experienced higher mental distress than caregivers who have good social support therefore every possible action should be taken to expand social support. The study results also suggest that researchers and mental health professionals should attend to the special needs of caregivers who are female, farmer, have lower levels of education, and caregivers who took care for patients with psychotic disorder and patients with multiple admissions at risk of mental distress. Generally these findings called for measures to be taken to ensure that the needs of caregivers are met.

### Recommendations

Interventions could be developed that have a focus on the health and well-being of the caregivers for instance, interventions that focus on stress reduction and health promotion activities.

Scheduled and ongoing psycho-education and mutual support programme could be implemented and strengthened that helps to cope stress, empowering caregivers with knowledge and develop their competence in handling illness of care recipient and enhance their chance of living a life that is as normal as possible. Because this psycho-education and mutual support may compensate for deficiencies in people’s natural support networks.

Moreover, it suggest that rather than exclusively targeting patients’ treatments, mental distress screenings and emotional health assessments for diagnosis of probable mental distress could be conducted for caregivers and is likely to yield significant payoffs in terms of reducing caregivers’ mental distress.

Efforts should be exerted for treating patients effectively to minimize relapse or frequent admission as much possible.

Future work should be directed at specific mental disorders by further characterizing the nature and severity of illness.

## Abbreviations

AOR: Adjusted Odds Ratio
CES-D: Center for Epidemiologic Studies-Depression Scale
COR: Crude Odds Ratio
GHQ: General Health Questionnaire
OR: Odds Ratio
SPSS: Statistical Package for Social Science
SRQ: Self Reported Questionnaire
WFHO: World Federation Health Organization
WHO: World Health Organization
